# Impact of gastrointestinal multiplex PCR on the diagnosis, clinical correlation, and antimicrobial stewardship in pediatric acute gastroenteritis

**DOI:** 10.3389/fped.2026.1735228

**Published:** 2026-04-28

**Authors:** Afnan AlHazimi, Areej AlQahtani, Afnan AlSahli, Amal AlOtaibi, Maha Sheikho, Reema AlZighaibi, Bedoor Al-Qadrah

**Affiliations:** 1 Department of Pediatrics, King Abdullah Specialized Children’s Hospital, King Abdulaziz Medical City, Ministry of National Guard Health Affairs, Riyadh, Saudi Arabia; 2 King Abdullah International Medical Research Center, Ministry of National Guard Health Affairs, Riyadh, Saudi Arabia; 3 Ministry of National Guard Health Affairs, Riyadh, Saudi Arabia

**Keywords:** acute gastroenteritis, antibiotic stewardship, gastrointestinal multiplex PCR, pathogen detection, pediatric infections, Saudi Arabia, stool culture, viral and bacterial gastroenteritis

## Abstract

**Introduction:**

Acute gastroenteritis (AGE) is a major cause of childhood morbidity and mortality worldwide, yet pathogen identification often remains elusive. Gastrointestinal (GI) multiplex polymerase chain reaction (PCR) enables simultaneous detection of multiple enteropathogens with greater accuracy and faster turnaround than conventional methods. This study aimed to assess the diagnostic and clinical impact of GI multiplex PCR testing on pediatric gastroenteritis at a tertiary care hospital in Saudi Arabia.

**Methods:**

A retrospective observational study was conducted at King Abdullah Specialized Children's Hospital, Riyadh, including all children aged ≤14 years admitted with gastroenteritis who underwent GI multiplex PCR testing between January 2022 and December 2024. Clinical data, laboratory results, and management outcomes were retrieved from electronic medical records. Stool samples were analyzed using the BioFire FilmArray GI panel, with stool culture and ova/parasite testing performed as indicated.

**Results:**

A total of 276 children were included, with infants aged 1–23 months comprising the largest subgroup (43.5%). GI multiplex PCR was positive in 90.9% of cases, with single-pathogen detections in 56.2% and co-detections in 34.8%. The most common pathogens were Salmonella (22.1%), Rotavirus A (18.1%), Norovirus (17.4%), and Clostridium difficile (17.0%). Antibiotic use differed by pathogen type, being lower in viral infections (27.4%) and higher in bacterial infections (45.3%). Following PCR results, antibiotics were initiated in 41.3% of bacterial cases and discontinued in 39.4% of viral cases. Detection of bacterial pathogens and abnormal white blood cell count were independently associated with antibiotic use. Inflammatory markers, particularly ESR, were higher in bacterial infections, while length of hospital stay did not differ significantly by pathogen type.

**Conclusion:**

GI multiplex PCR demonstrated high diagnostic yield and was associated with differences in antibiotic use according to pathogen type, suggesting a potential role in informing targeted therapy and antimicrobial stewardship in pediatric AGE. However, interpretation requires clinical correlation, particularly in cases of co-detection and pathogens with known asymptomatic carriage. Conventional stool culture remains complementary for bacterial isolation and susceptibility testing.

## Introduction

Acute Gastroenteritis (AGE) is a common infectious disease among infants and children. It is implicated in a high percentage of morbidity and mortality throughout the world, especially among children <5 years, with an estimated mortality of 2 million children annually (1). Multiple microorganisms, including viruses, bacteria, and protozoan parasites, can cause AGE (2, 3). The etiology remained unidentified in 80% of patients with AGE (4). Pathogen identification can be a crucial guide for patient management, the choice of antimicrobial treatment, and the avoidance of inappropriate antibiotic use, as well as the prevention of further evolution of antimicrobial resistance (5). Traditional methods for identifying AGE pathogens include stool cultures, staining or ova and parasites, and single antigen detection methods (2). These methods require more time, effort, and cost when compared to new diagnostic techniques. One newly implemented method for detecting infectious GI pathogens is the GI multiplex PCR assay, which enables the simultaneous detection of specific bacterial, viral, and parasitic targets (5). This test is superior to traditional diagnostic tests in terms of accuracy, ease of use, and evaluation of multiple enteropathogens by a single assay (5). GI multiplex PCR offers the benefits of rapid, timely diagnosis and the avoidance of antibiotic over prescription, side effects, and resistance (2). A study done in India included 414 patients using GI multiplex PCR and found that antibiotics were used in 90% of patients with viral AGE (2). In low- and middle-income countries, the Global Enteric Multicenter Study (GEMS) demonstrated that bacterial pathogens, including Shigella, enterotoxigenic Escherichia coli, and Campylobacter, remain major causes of moderate-to-severe pediatric diarrhea. In a separate multicountry analysis, children under five were shown to experience substantial antibiotic exposure. Multiplex PCR has been shown to improve pathogen detection and may facilitate more targeted antimicrobial use, thereby supporting antimicrobial stewardship efforts (2, 6, 7). Multiple factors impede the implementation of GI multiplex PCR, including cost-effectiveness, the need for specialized and sophisticated equipment, the risk of overdiagnosis, and the lack of evidence-based clinical criteria for its use. Freeman et al. found that a high number of favorable results were observed with multiplex testing compared to conventional testing (2). The significance of these positive results for guiding patients' management remains unclear and lacks evidence. Furthermore, in clinical practice, universal testing for all patients presenting with AGE is not recommended, as the most common cause is viral, and to date, there are no approved antimicrobial recommendations for such cases. However, it is important to recognize common viruses to help implement or improve preventive measures that reduce the disease burden, such as Rotavirus vaccines (2). Emerging evidence supports the benefits of GI multiplex testing in the treatment of AGE. A study conducted at a tertiary care center in Gainesville, Florida, USA, using the BioFire FilmArray GI PCR panel in a mixed adult and pediatric population demonstrated reduced abdominal/pelvic imaging (0.39–0.18 per patient), fewer additional stool tests (3.02–0.58 per patient), and shorter antibiotic duration (2.21–1.73 days) (8). Additionally, Cybulski et al. reported that implementation reduced antibiotic duration from 72 to 26 h and decreased empiric antimicrobial use from 40% to 23.5% (9). However, studies evaluating the impact of GI multiplex PCR panels on antibiotic utilization have reported heterogeneous findings. For example, in hospitalized adults, implementation of GI PCR testing improved pathogen detection but was not consistently associated with reduced antibiotic exposure (10). Furthermore, systematic reviews indicate that robust evidence demonstrating consistent improvement in downstream clinical outcomes remains limited (11).

This study aims to assess the role of GI multiplex PCR in diagnosing and managing pediatric AGE, with the intention of defining its diagnostic utility and guiding clinical practice.

## Methodology

This retrospective observational study was conducted at King Abdullah Specialized Children's Hospital (KASCH), a 600-bed tertiary pediatric center within King Abdulaziz Medical City in Riyadh, Saudi Arabia. The study included all children aged 14 years or younger who were admitted with a clinical diagnosis of gastroenteritis and underwent GI multiplex PCR testing between January 2022 and December 2024. Data were extracted from the BestCare electronic medical records using a standardized chart review form. Collected variables included demographic characteristics, clinical presentation, laboratory investigations, PCR results, and clinical management. The primary outcome of the study was the diagnostic yield of GI multiplex PCR in identifying pathogens causing AGE. Secondary outcomes included changes in clinical management following PCR results (such as antibiotic initiation, continuation, or discontinuation), length of hospital stay, and other patient outcomes during hospitalization.

Following PCR results. Stool samples were analyzed using the BioFire FilmArray GI panel (BioFire Diagnostics, Salt Lake City, UT, USA), with additional stool cultures and ova and parasite testing performed as clinically indicated. The BioFire FilmArray GI panel includes five viruses, four parasites, and 13 bacteria commonly associated with gastroenteritis (Table 1).

**Table 1:** Biofire filmArray GI panel pathogens.

Viral pathogen	Bacterial pathogen	Diarrheagenic E. Coli/shigella	Parasitic pathogen
Sapovirus	Clostridium difficile toxin A/B	Enteropathogenic E. coli	Cryptosporidium
Adenovirus F 40/41	Campylobacter	Enterotoxigenic E. coli	Cyclospora cayetanensis
Rotavirus A	Plesiomonas shigelloides	Shiga-like toxin-producing E. coli	Entamoeba histolytica
Norovirus GI/GII	Salmonella	E. coli O157	Giardia lamblia
Astrovirus	Vibrio	Shigella/Enteroinvasive E. coli	
	Vibrio cholerae	Enteroaggregative E. coli	
	Yersinia enterocolitica		

Inclusion criteria encompassed patients who met the diagnostic criteria for AGE, defined as the passage of three or more loose stools within 24 h, with or without vomiting or dehydration, and who had stool samples analyzed using the GI multiplex PCR panel during the study period. Only hospitalized patients were included in the analysis. Patients evaluated in the emergency department or outpatient settings who were not subsequently admitted were excluded. Patients were also excluded if they were older than 14 years, had incomplete medical records, underwent stool testing by non-PCR methods, or had underlying chronic GI diseases that could independently cause chronic diarrhea or alter intestinal microbiology, including inflammatory bowel disease, celiac disease, short bowel syndrome, congenital or anatomical GI abnormalities, and chronic malabsorption disorders.

At our institution, there is no formal institutional algorithm guiding GI multiplex PCR testing. The decision to order the test is based on the treating physician's clinical judgment for hospitalized pediatric patients presenting with suspected acute gastroenteritis when pathogen identification may influence clinical management.

Ethical approval was granted by the Institutional Review Board of King Abdullah International Medical Research Center (IRP number: NRR24/130/9), with informed consent waived due to the retrospective design. Patients' confidentiality was maintained by assigning study codes in place of identifiers, and all data were securely stored with access restricted to the study team.

### Statistical analysis

Data were analyzed using RStudio (version 2024.9.1.394, Boston, MA, USA) with R version 4.4.2. Descriptive statistics were used to summarize demographic and clinical variables, reported as frequencies and percentages. Associations between categorical variables were assessed using Pearson's Chi-squared test or Fisher's exact test, as appropriate based on cell counts. Comparisons of outcomes across age groups, GI PCR results, symptom profiles, antibiotic usage patterns, and laboratory findings were conducted using these tests. A two-sided *p*-value of less than 0.05 was considered statistically significant for all analyses.

Missing data were handled using a complete-case approach. For each variable, analyses were performed using the available data, and denominators were adjusted accordingly.

Co-detections were defined as the identification of more than one pathogen in the same stool sample using the GI multiplex PCR panel. For analytic purposes, infections were categorized according to pathogen type (viral, bacterial, or parasitic). In samples with multiple pathogens detected, cases containing any bacterial pathogen were classified within the bacterial group for comparative analyses due to their potential clinical relevance for antimicrobial therapy. Viral-only and parasitic-only detections were categorized accordingly.

## Result

A total of 276 pediatric patients were analyzed, with infants aged 1–23 months comprising the largest subgroup (43.5%). Acute gastroenteritis was the predominant diagnosis (97.5%), with fever occurring in 76.4% of cases and diarrhea in 99.3% of cases, most often lasting for ≤2 days (52.7%) (Table 2).

**Table 2:** Demographic and clinical characteristics of patients.

Characteristic	Description
Age	
1–23 months	120/276 (43.5%)
2–4 yrs	93/276 (33.7%)
5–14 yrs4	63/276 (22.8%)
Gender	
Female	127/276 (46.0%)
Male	149/276 (54.0%)
Indication for hospitalization: age	
No	7/276 (2.5%)
Yes	269/276 (97.5%)
Indication for hospitalization: other reason	
Provoked Seizure	1/276 (0.4%)
other indication	269/276 (97.5%)
Meningitis	2/276 (0.7%)
Pneumonia	1/276 (0.4%)
Bloody stool for evaluation	1/276 (0.4%)
Neutropenia	0/276 (0.0%)
Acute otitis media	1/276 (0.4%)
Bacteremia	1/276 (0.4%)
Fever	
No	65/276 (23.6%)
Yes	211/276 (76.4%)
If Yes (Specify)	
≤2 days	125/276 (45.3%)
3–5 Days	69/276 (25.0%)
≥6 days.	17/276 (6.2%)
N/A	65/276 (23.6%)
Diarrhea	
No	2/276 (0.7%)
Yes	274/276 (99.3%)
If Yes (Specify)	
≤2 days	145/275 (52.7%)
3–5 Days	88/275 (32.0%)
≥6 days.	40/275 (14.5%)
N/A	2/275 (0.7%)
Unknown	1/276 (0.4%)
Bloody diarrhea	
No	237/276 (85.9%)
Yes	39/276 (14.1%)
Vomiting	
No	55/276 (19.9%)
Yes	221/276 (80.1%)
If Yes (Specify)	
≤2 days	124/221 (56.1%)
3–5 Days	79/221 (35.7%)
≥6 days.	18/221 (8.1%)
Unknown	55/276 (19.9%)
Abdominal pain	
No	169/276 (61.2%)
Yes	52/276 (18.8%)
N/A	55/276 (19.9%)
If Yes (Specify)	
≤2 days	24/276 (8.7%)
3–5 Days	24/276 (8.7%)
≥6 days.	9/276 (3.3%)
N/A	219/276 (79.3%)
Respiratory symptoms	
No	231/276 (83.7%)
Yes	45/276 (16.3%)
History of eating from outside (suspected food poisoning)	
No	39/276 (14.1%)
Yes	17/276 (6.2%)
Unknown	220/276 (79.7%)

n/N (%).

Symptom distribution demonstrated significant age-related variation. Vomiting was most prevalent among toddlers aged 2–4 years (88.2%), compared with infants (71.7%), and older children aged 5–14 years (84.1%) (*p* = 0.007). Abdominal pain also showed an age-dependent pattern, being most frequent in children aged 5–14 years (57.1%; *p* < 0.001). This finding however should be interpreted cautiously in younger age groups, as abdominal pain may be underreported in infants who cannot reliably communicate pain symptoms. No significant gender-related differences were identified for any symptom (all *p* > 0.05). In relation to clinical presentation and antibiotic exposure, fever was more frequently observed in patients who received antibiotics (83.5% vs. 72.3%, *p* = 0.033), and bloody diarrhea was likewise more common in this group (20.4% vs. 10.4%, *p* = 0.021).

The overall GI multiplex PCR positivity rate was 251/276 (90.9%, 95% CI 87.0–93.8). Among the study population, single-pathogen infections occurred in 56.2% of cases, while co-detections were observed in 34.8%. Dual-pathogen detections accounted for 23.9% of cases, and three or more pathogens were identified in 10.9%, whereas 9.1% of samples were negative. Among patients with multiple pathogens detected (*n* = 96), the most frequent combination was viral–bacterial co-detection (56.3%), followed by bacterial–bacterial detections (32.3%). Viral–viral co-detections accounted for 5.2% of cases, while viral–parasitic and bacterial–parasitic combinations were each observed in 3.1% of cases (Table 3).

**Table 3:** Frequencies of positive infections, mixed detections, and E. coli pathotype distribution.

Characteristic	Description
Detection category	
None	25/276 (9.1%)
Single Pathogen	155/276 (56.2%)
Dual Pathogens	66/276 (23.9%)
≥3 Pathogens	30/276 (10.9%)
Mixed detection category	
Bacterial + Bacterial	31/96 (32.3%)
Bacterial + Parasitic	3/96 (3.1%)
Viral + Bacterial	54/96 (56.3%)
Viral + Parasitic	3/96 (3.1%)
Viral + Viral	5/96 (5.2%)
E. coli pathotype distribution	
EnteroAggregative E coli (EAEC)	26/276 (9.4%)
Enteropathogenic E coli (EPEC)	44/276 (15.9%)
EnterotoxIgenic E coli (ETEC) lt/st	9/276 (3.3%)
Shiga-like toxin-producing E coli (STEC) stx1/stx2	4/276 (1.4%)
E.coli O157	1/276 (0.4%)
ShIgella/Enteroinvasive E coli (EIEC)	12/276 (4.3%)

n/N (%).

The most frequently detected pathogens were Salmonella in 22.1% of patients, Norovirus GI/GII in 17.4%, Rotavirus A in 18.1%, and Clostridium difficile (C. difficile) toxin A/B in 17.0%. C. difficile positivity did not differ significantly between age groups (19.2% in children <2 years vs. 15.4% in those ≥2 years, *p* = 0.407).

Stool cultures were not obtained in 56.9% of cases; among those performed, 32.2% yielded negative results, 9.8% confirmed *Salmonella*, and less frequent findings included *Campylobacter jejuni* (0.7%) and *Shigella* species (0.4%). Ova and parasite examinations were omitted in 73.9% of patients, and all completed tests were negative. Viral pathogen detection by GI multiplex PCR was significantly associated with stool culture results (*p* = 0.007), with negative cultures more frequently observed among viral-positive cases compared with viral-negative cases (84.2% vs. 70.4%). Similarly, bacterial pathogen detection was significantly associated with culture findings (*p* = 0.001), with *Salmonella* isolated in 32.1% of bacterial-positive cases vs. 4.9% of bacterial-negative cases. In contrast, parasitic pathogen detection showed no significant association with either stool culture or ova and parasite testing (Table 4). Concordance analysis between multiplex PCR and stool culture for selected bacterial pathogens demonstrated that stool culture confirmed *Salmonella* in 24 of 61 cases (39.3%), *Campylobacter* in 2 of 25 cases (8.0%), and *Shigella* in 1 of 12 cases (8.3%).

**Table 4:** Clinical and laboratory characteristics associated with viral, bacterial, and parasitic detections by GI multiplex PCR.

Characteristic	Viral pathogen			Bacterial pathogen			Parasitic pathogen		
	No *N* = 81^a^	Yes *N* = 38^a^	*p*-value^b^	No *N* = 41^a^	Yes *N* = 78^a^	*p*-value^b^	No *N* = 114^a^	Yes *N* = 5^a^	*p*-value^b^
Stool culture			0.007			<0.001			0.639
Negative	57/81 (70.4%)	32/38 (84.2%)		39/41 (95.1%)	50/78 (64.1%)		84/114 (73.7%)	5/5 (100.0%)	
Salmonella	21/81 (25.9%)	6/38 (15.8%)		2/41 (4.9%)	25/78 (32.1%)		27/114 (23.7%)	0/5 (0.0%)	
Campylobacter jejuni	2/81 (2.5%)	0/38 (0.0%)		0/41 (0.0%)	2/78 (2.6%)		2/114 (1.8%)	0/5 (0.0%)	
Shigella species	1/81 (1.2%)	0/38 (0.0%)		0/41 (0.0%)	1/78 (1.3%)		1/114 (0.9%)	0/5 (0.0%)	
Ova and parasite examination			0.039			0.261			>0.999
Negative	49/159 (30.8%)	23/117 (19.7%)		23/104 (22.1%)	49/172 (28.5%)		69/264 (26.1%)	3/12 (25.0%)	
Positive (Specify)	0/159 (0.0%)	0/117 (0.0%)		0/104 (0.0%)	0/172 (0.0%)		0/264 (0.0%)	0/12 (0.0%)	
Not sent	110/159 (69.2%)	94/117 (80.3%)		81/104 (77.9%)	123/172 (71.5%)		195/264 (73.9%)	9/12 (75.0%)	
Fever			0.323			0.013			0.161
No	34/159 (21.4%)	31/117 (26.5%)		33/104 (31.7%)	32/172 (18.6%)		60/264 (22.7%)	5/12 (41.7%)	
Yes	125/159 (78.6%)	86/117 (73.5%)		71/104 (68.3%)	140/172 (81.4%)		204/264 (77.3%)	7/12 (58.3%)	
Diarrhea			>0.999			>0.999			>0.999
No	1/159 (0.6%)	1/117 (0.9%)		1/104 (1.0%)	1/172 (0.6%)		2/264 (0.8%)	0/12 (0.0%)	
Yes	158/159 (99.4%)	116/117 (99.1%)		103/104 (99.0%)	171/172 (99.4%)		262/264 (99.2%)	12/12 (100.0%)	
Bloody diarrhea			<0.001			<0.001			>0.999
No	127/159 (79.9%)	110/117 (94.0%)		99/104 (95.2%)	138/172 (80.2%)		226/264 (85.6%)	11/12 (91.7%)	
Yes	32/159 (20.1%)	7/117 (6.0%)		5/104 (4.8%)	34/172 (19.8%)		38/264 (14.4%)	1/12 (8.3%)	
Vomiting			0.011			0.184			0.133
No	40/159 (25.2%)	15/117 (12.8%)		25/104 (24.0%)	30/172 (17.4%)		55/264 (20.8%)	0/12 (0.0%)	
Yes	119/159 (74.8%)	102/117 (87.2%)		79/104 (76.0%)	142/172 (82.6%)		209/264 (79.2%)	12/12 (100.0%)	
Abdominal pain			<0.001			0.143			0.333
No	88/159 (55.3%)	81/117 (69.2%)		63/104 (60.6%)	106/172 (61.6%)		162/264 (61.4%)	7/12 (58.3%)	
Yes	42/159 (26.4%)	10/117 (8.5%)		15/104 (14.4%)	37/172 (21.5%)		48/264 (18.2%)	4/12 (33.3%)	
N/A	29/159 (18.2%)	26/117 (22.2%)		26/104 (25.0%)	29/172 (16.9%)		54/264 (20.5%)	1/12 (8.3%)	
Respiratory symptoms			0.335			0.018			0.697
No	136/159 (85.5%)	95/117 (81.2%)		80/104 (76.9%)	151/172 (87.8%)		220/264 (83.3%)	11/12 (91.7%)	
Yes	23/159 (14.5%)	22/117 (18.8%)		24/104 (23.1%)	21/172 (12.2%)		44/264 (16.7%)	1/12 (8.3%)	

a n/N (%).

b Fisher’s exact test.

Bloody diarrhea was significantly more common in patients with viral-negative compared to viral-positive PCR (20.1% vs. 6.0%, *p* < 0.001) and in patients with bacterial-positive compared to bacterial-negative PCR (19.8% vs. 4.8%, *p* < 0.001). Vomiting was more frequent among patients with viral pathogens (87.2% vs. 74.8%, *p* = 0.011), while fever was more often observed in those with bacterial pathogens (81.4% vs. 68.3%, *p* = 0.013). Conversely, respiratory symptoms were less frequent in bacterial infections (12.2% vs. 23.1%, *p* = 0.018). No significant associations were found between clinical symptoms and parasitic pathogen detection (all *p* > 0.05) (Table 4).

To further evaluate the clinical relevance of laboratory markers, analyses were performed according to pathogen type. WBC count and platelet levels did not differ significantly across groups of pathogen category (*p* = 0.837 and *p* = 0.298, respectively). In contrast, ESR differed significantly by pathogen category (*p* < 0.001), with abnormal ESR more frequently observed in bacterial-only infections (59.5%) compared with viral-only (7.1%), parasitic-only (0.0%), and mixed infections (50.0%). CRP levels showed a non-significant trend toward higher abnormal values in bacterial infections (73.8%) compared with viral (35.7%), parasitic (50.0%), and mixed infections (62.5%) (*p* = 0.055).

Antibiotics were prescribed in 37.3% of patients, with most initiated before GI multiplex PCR results were available (65.0%) and the remainder afterward (35.0%). Cephalosporins were the predominant class used (66.0%), followed by macrolides (14.9%) and nitroimidazoles (13.8%). Treatment duration was generally short, with 70.6% of courses lasting <7 days. Antibiotic use in comparisons across pathogen groups refers to total antibiotic exposure during hospitalization, including antibiotics initiated both before and after PCR results. Using this definition, antibiotic utilization differed significantly by pathogen type, being less frequent in viral infections (27.4% vs. 44.7%, *p* = 0.003) and more frequent in bacterial infections (45.3% vs. 24.0%, *p* < 0.001). The timing of antibiotic initiation also reflected pathogen status: all pathogen-negative patients who received antibiotics were started before PCR results were available, whereas 46.2% of bacterial-positive cases were initiated after PCR results (*p* < 0.001). Following PCR, antibiotics were initiated in 41.3% of bacterial cases but in none of the pathogen-negative cases, while discontinuation was more common in viral compared with non-viral infections (39.4% vs. 17.8%, *p* = 0.036). Antibiotic duration was significantly longer in bacterial (35.1%) and parasitic infections (100%) compared with their counterparts (8.3% and 25.8%, respectively; *p* = 0.011 and *p* < 0.001). No significant associations were observed between pathogen type and hospital length of stay (Table 5).

**Table 5:** Correlation between pathogen types and the patterns of antibiotics use.

Characteristic	Viral pathogen			Bacterial pathogen			Parasitic pathogen		
	No *N* = 159^a^	Yes *N* = 117^a^	*p*-value^b^	No *N* = 104^a^	Yes *N* = 172^a^	*p*-value^b^	No *N* = 264^a^	Yes *N* = 12^a^	*p*-value^c^
Antibiotics used	71/159 (44.7%)	32/117 (27.4%)	0.003	25/104 (24.0%)	78/172 (45.3%)	<0.001	99/264 (37.5%)	4/12 (33.3%)	>0.999
If antibiotic, started before or after GI PCR result			0.155			<0.001			0.610
Before result	43/71 (60.6%)	24/32 (75.0%)		25/25 (100.0%)	42/78 (53.8%)		65/99 (65.7%)	2/4 (50.0%)	
After result	28/71 (39.4%)	8/32 (25.0%)		0/25 (0.0%)	36/78 (46.2%)		34/99 (34.3%)	2/4 (50.0%)	
Duration of antibiotic			0.768			0.011			0.006
Less than 7 days	50/71 (70.4%)	22/30 (73.3%)		22/24 (91.7%)	50/77 (64.9%)		72/97 (74.2%)	0/4 (0.0%)	
More than 7 days	21/71 (29.6%)	8/30 (26.7%)		2/24 (8.3%)	27/77 (35.1%)		25/97 (25.8%)	4/4 (100.0%)	
Total Length of stay			0.779			0.489			0.151
<2 days	60/159 (37.7%)	45/116 (38.8%)		35/103 (34.0%)	70/172 (40.7%)		102/263 (38.8%)	3/12 (25.0%)	
3–5 Days	86/159 (54.1%)	59/116 (50.9%)		59/103 (57.3%)	86/172 (50.0%)		139/263 (52.9%)	6/12 (50.0%)	
>6 days	13/159 (8.2%)	12/116 (10.3%)		9/103 (8.7%)	16/172 (9.3%)		22/263 (8.4%)	3/12 (25.0%)	
Length of stay from PCR result till discharge			0.473			0.577			0.041
≤2 days	112/159 (70.4%)	87/117 (74.4%)		77/104 (74.0%)	122/172 (70.9%)		194/264 (73.5%)	5/12 (41.7%)	
3 days or more	47/159 (29.6%)	30/117 (25.6%)		27/104 (26.0%)	50/172 (29.1%)		70/264 (26.5%)	7/12 (58.3%)	
Treatment decision changes after GI PCR result			0.036			<0.001			0.681
Continue Antibiotic	20/73 (27.4%)	11/33 (33.3%)		12/26 (46.2%)	19/80 (23.8%)		30/102 (29.4%)	1/4 (25.0%)	
Start Antibiotic	26/73 (35.6%)	7/33 (21.2%)		0/26 (0.0%)	33/80 (41.3%)		31/102 (30.4%)	2/4 (50.0%)	
Change Antibiotic	14/73 (19.2%)	2/33 (6.1%)		1/26 (3.8%)	15/80 (18.8%)		15/102 (14.7%)	1/4 (25.0%)	
Discontinue antibiotic	13/73 (17.8%)	13/33 (39.4%)		13/26 (50.0%)	13/80 (16.3%)		26/102 (25.5%)	0/4 (0.0%)	

a n/N (%).

b Pearson’s Chi-squared test.

c Fisher’s exact test.

A supplementary analysis examined viral-only cases receiving antibiotics (Table 6). Among viral-only infections, 14 of 70 patients (20.0%) received antibiotics. Most had single-pathogen viral detections (13/14, 92.9%), and none had positive blood or urine cultures, suggesting that antibiotic use in these cases likely reflected empiric clinical decision-making rather than confirmed bacterial co-infection.

**Table 6:** Detailed stewardship analysis and characteristics of viral-only cases receiving antibiotics.

Characteristics	Description
Antibiotics started before PCR (among patients receiving antibiotics)	67/103 (65.0%)
Antibiotics discontinued after PCR (among patients who started antibiotics before PCR)	25/67 (37.3%)
Antibiotics started after PCR (among patients receiving antibiotics)	36/103 (35.0%)
Viral-only cases receiving antibiotics (among viral-only infections)	14/70 (20.0%)
Detection category	
Single	13/14 (92.9%)
Dual	1/14 (7.1%)
Any positive blood or urine culture	
Negative	14/14 (100.0%)

n/N (%).

To further evaluate factors associated with antibiotic prescribing, a multivariable logistic regression analysis was performed including age, fever, bloody diarrhea, inflammatory markers, and PCR pathogen detection. Abnormal white blood cell count was independently associated with increased odds of antibiotic prescribing (adjusted OR 2.23, 95% CI 1.25–4.00, *p* = 0.007). Detection of bacterial pathogens by GI multiplex PCR was also independently associated with antibiotic use (adjusted OR 2.19, 95% CI 1.16–4.23, *p* = 0.017). Other variables, including age, fever, bloody diarrhea, CRP levels, viral pathogen detection, and parasitic pathogen detection, were not significantly associated with antibiotic prescribing after adjustment.

## Discussion

This study evaluated the clinical and diagnostic implications of gastrointestinal (GI) multiplex PCR testing in hospitalized pediatric patients with acute gastroenteritis (AGE) at a tertiary care center. Infants aged 1–23 months constituted the largest subgroup, and symptom patterns varied across age groups, with vomiting predominating in younger children and abdominal pain more frequently reported in older children. Clinical features suggestive of invasive disease, including fever and bloody diarrhea, were more commonly observed in patients with bacterial pathogen detection. These findings are consistent with prior literature indicating that viral AGE is often associated with vomiting, whereas bacterial infections are more likely to present with inflammatory features such as fever and bloody diarrhea (12–16). Collectively, these observations reinforce the role of clinical presentation in guiding initial diagnostic suspicion, particularly in settings where immediate molecular testing may not be available.

Laboratory markers varied according to pathogen type, with higher levels, particularly ESR, were observed in bacterial infections compared with viral infections. This pattern is consistent with the inflammatory nature of invasive bacterial gastroenteritis, as bacterial pathogens such as *Salmonella* and *Shigella* are known to elicit higher fevers and elevated inflammatory markers compared with viral infections (12, 17). However, in our cohort, CRP level demonstrated a similar but non-significant trend, while WBC and platelet count did not differ significantly across different groups of pathogen category. These findings partially support existing evidence that inflammatory markers may aid in distinguishing bacterial from non-bacterial gastroenteritis. Previous studies have reported that CRP thresholds (e.g., ≥2.3 mg/dL) can help differentiate bacterial from viral infections with good diagnostic performance (16). However, the discriminatory value of inflammatory markers remains limited when used in isolation and should be interpreted in conjunction with the overall clinical context.

The overall PCR positivity rate in this study was high (90.9%), which likely reflects multiple contributing factors. First, the study population consisted exclusively of hospitalized children in a tertiary care setting, representing a preselected cohort. Second, testing was performed at the discretion of treating physicians, introducing potential selection bias toward patients in whom pathogen identification was deemed clinically relevant. Third, the substantial proportion of co-detections (34.8%) contributed to increased overall positivity. Finally, the high analytical sensitivity of multiplex PCR assays allows detection of low-level nucleic acid, which may represent residual shedding or colonization rather than active infection, particularly in younger children (18).

Differences between conventional stool culture and molecular testing were also evident. Although stool cultures were performed in only approximately 43% of patients, a significant association was observed between GI multiplex PCR results and stool culture findings among those tested. In our cohort, bacterial pathogen detection by PCR showed concordance with stool culture positivity, whereas viral-positive cases were more frequently associated with negative stool cultures, reflecting the limited ability of conventional methods to detect nonbacterial pathogens. These findings align with prior studies demonstrating that traditional diagnostic methods may miss a substantial proportion of pathogens identified by multiplex PCR panels. For example, the BioFire GI panel has been shown to achieve higher detection rates compared with conventional testing (58.3% vs. 42.3%) and historical culture-based cohorts (31.4%) (8). Similarly, multiplex PCR implementation has been associated with rapid pathogen identification in over 90% of cases within 48 h of symptom onset (19).

Multiplex PCR assays offer faster and more sensitive detection of a broad range of pathogens, often within 24 h, which may support more timely and targeted clinical decision-making and improved infection control practices. However, stool culture remains essential for antimicrobial susceptibility testing and epidemiologic surveillance, particularly for bacterial pathogens (18).

The high rate of co-detections observed in this study represents a key diagnostic and interpretative challenge associated with multiplex molecular testing. Approximately one-third of patients had more than one pathogen detected, most commonly viral–bacterial combinations. While such findings may reflect true polymicrobial infections, they may also represent coincidental detection of colonizing organisms or prolonged shedding following recent infection. Interpretation of multiplex PCR results therefore requires careful clinical correlation because these assays detect nucleic acids rather than viable organisms and may identify pathogens of uncertain clinical relevance (5, 18). For example, detection of certain organisms, including enteroaggregative and enteropathogenic *Escherichia coli*, are frequently identified in asymptomatic carriers. Failure to recognize this distinction may lead to unnecessary antimicrobial therapy and inappropriate clinical management (20). Another example is Clostridium difficile (C. difficile) which was detected in a subset of patients in our cohort. Its detection, particularly in children under 2 years of age, should also be interpreted with caution, as asymptomatic colonization is common in this age group and may not reflect true infection (21).

With respect to antimicrobial stewardship, our findings suggest that GI multiplex PCR testing was associated with differences in antibiotic prescribing patterns. Antibiotic use was less frequent in viral infections and more common in cases with bacterial pathogen detection. In addition, antibiotics were discontinued more frequently following viral identification, whereas initiation after PCR results was more common in bacterial cases. However, these findings should be interpreted cautiously, as the observational design precludes inference of causality. Antibiotic decisions are influenced by multiple factors, including clinical severity, physician judgment, and institutional practices, and PCR results likely represent one component of a broader decision-making process. Notably, a subset of viral-only cases still received antibiotics, reflecting the persistence of empiric prescribing in the absence of definitive exclusion of bacterial infection. These findings are consistent with prior studies demonstrating that implementation of multiplex PCR testing may reduce antibiotic use; for example, one study reported a decrease from 71.8% to 35.3% following introduction of a multiplex gastrointestinal panel (19). In contrast, other studies have reported that although multiplex testing improves diagnostic speed, it does not substantially alter antibiotic prescribing or overall management (22). Collectively, these findings suggest that GI multiplex PCR may help inform antibiotic decisions and reduce antimicrobial exposure in tertiary pediatric settings.

Furthermore, this study demonstrated no significant association between GI multiplex PCR results and hospital length of stay. This may reflect the multifactorial determinants of hospitalization in pediatric acute gastroenteritis, including dehydration, electrolyte imbalance, and the need for supportive management. Additionally, evaluation for suspected invasive infections, such as bacteremia, may prolong admission regardless of pathogen detection. Previous studies have similarly shown that although multiplex PCR improves pathogen detection, its impact on downstream clinical outcomes remains uncertain (11). These findings suggest that while GI multiplex PCR enhances diagnostic clarity, it may not independently influence hospital length of stay.

Future research should refine interpretation frameworks for multiplex PCR and integrate them into clinical decision-support systems to strengthen antimicrobial stewardship. Moreover, Prospective and cost-effectiveness studies are warranted to assess the balance between diagnostic precision and resource use, as well as the impact on outcomes such as complication rates and antibiotic resistance. Such efforts will ensure that molecular diagnostics in pediatric gastroenteritis achieve their full potential in improving care quality and stewardship.

## Strength and limitations

This single-center retrospective design carries inherent limitations, including selection and verification bias due to clinician-directed, non-probability testing. Stool cultures and ova/parasite exams were inconsistently obtained, potentially affecting PCR–culture correlations. Analyses were mainly univariable, limiting causal inference, and multiplex panels may have detected colonization rather than true infection. Additionally, co-detections were not adjudicated, and outcome measures were limited to intermediate clinical endpoints such as antibiotic use.

Despite its limitations, this retrospective cohort included 276 pediatric inpatients admitted to a tertiary children's hospital between 2022 and 2024, providing a real-world, age-stratified population for potentially clinically meaningful analysis. The study integrated GI multiplex PCR, stool culture, enabling cross-method validation and linking diagnostic results to key antimicrobial stewardship outcomes such as antibiotic initiation, timing, and treatment duration.

## Conclusion

Rapid GI multiplex PCR was associated with higher etiologic detection rates and changes in management patterns in pediatric acute gastroenteritis. Viral detections were associated with lower antibiotic use, whereas bacterial identifications corresponded with more targeted antimicrobial therapy, with nearly half of antibiotic courses initiated following PCR results. Conventional stool culture remained complementary for bacterial isolation and antimicrobial susceptibility testing.

Interpretation of certain pathotypes with known asymptomatic carriage requires careful clinical correlation. Moreover, the presence of co-detections further highlights the need for cautious interpretation of multiplex PCR findings and supports the use of this diagnostic tool primarily in hospitalized or clinically severe cases where pathogen identification is likely to influence management.

Overall, this study highlights the ongoing need for clinical correlation, stewardship oversight, and diagnostic algorithms integrating symptomatology and epidemiological context with molecular tests findings.

## Data Availability

The original contributions presented in the study are included in the article/Supplementary Material, further inquiries can be directed to the corresponding author.

## References

[1] King CK Glass R Bresee JS Duggan C, Centers for Disease Control and Prevention. Managing acute gastroenteritis among children: oral rehydration, maintenance, and nutritional therapy. MMWR Recommen Rep. (2003) 52(RR-16):1–16. 14627948

[2] Ghoshal U Tejan N. Rationale of using multiplex polymerase chain reaction (PCR) panels for etiological diagnosis of infective diarrhea in the tropics. Ind J Gastroenterol Off J Ind Soc Gastroenterol. (2018) 37(5):381–4. 10.1007/s12664-018-0908-z 30406887

[3] GBD 2016 Diarrhoeal Disease Collaborators. Estimates of the global, regional, and national morbidity, mortality, and aetiologies of diarrhoeal diseases: a systematic analysis for the Global Burden of Disease Study 2016. Lancet Infect Dis. (2018) 18(11):1211–28. 10.1016/S1473-3099(18)30362-1 30243583 PMC6202444

[4] Vernacchio L Vezina RM Mitchell AA Lesko SM Plaut AG Acheson DW. Diarrhea in American infants and young children in the community setting: incidence, clinical presentation and microbiology. Pediatr Infect Dis J. (2006) 25:2–7. 10.1097/01.inf.0000195623.57945.87 16395094

[5] Teh R Tee WD Tan E Fan K Koh CJ Tambyah PA Review of the role of gastrointestinal multiplex polymerase chain reaction in the management of diarrheal illness. J Gastroenterol Hepatol. (2021) 36(12):3286–97. 10.1111/jgh.15581 34129249

[6] Kotloff KL Nataro JP Blackwelder WC Nasrin D Farag TH Panchalingam S Burden and aetiology of diarrhoeal disease in infants and young children in developing countries (the Global Enteric Multicenter Study, GEMS): a prospective, case-control study. Lancet. (2013) 382(9888):209–22. 10.1016/S0140-6736(13)60844-2 23680352

[7] Fink G D'Acremont V Leslie HH Cohen J. Antibiotic exposure among children younger than 5 years in low-income and middle-income countries: a cross-sectional study of nationally representative facility-based and household-based surveys. Lancet Infect Dis. (2020) 20(2):179–87. 10.1016/S1473-3099(19)30572-9 31843383

[8] Beal SG Tremblay EE Toffel S Velez L Rand KH. A gastrointestinal PCR panel improves clinical management and lowers health care costs. J Clin Microbiol. (2018) 56(1):e01457–17. 10.1128/JCM.01457-17 29093106 PMC5744222

[9] Cybulski RJ Bateman AC Bourassa L Bryan A Beail B Matsumoto J Clinical impact of a multiplex gastrointestinal polymerase chain reaction panel in patients with acute gastroenteritis. Clin Infect Dis. (2018) 67(11):1688–96. 10.1093/cid/ciy357. 29697761

[10] Kelly GA Iordanov R Franklin A Ahmed A Srinivasan K Hayon J Impact of gastrointestinal polymerase chain reaction panels on antibiotic utilization in hospitalized adult patients. Antimicrob Steward Healthc Epidemiol. (2023) 3(1):e135. 10.1017/ash.2023.174 37592964 PMC10428147

[11] Freeman K Mistry H Tsertsvadze A Royle P McCarthy N Taylor-Phillips S Multiplex tests to identify gastrointestinal bacteria, viruses and parasites in people with suspected infectious gastroenteritis: a systematic review and economic analysis. Health Technol Assess. (2017) 21(23):1–188. 10.3310/hta21230 PMC5494512 28619124

[12] Harrison CJ Hassan F Lee B Boom J Sahni LC Johnson C Multiplex PCR pathogen detection in acute gastroenteritis among hospitalized US children compared with healthy controls during 2011–2016 in the post–rotavirus vaccine era. Open Forum Infect Dis. (2021) 8(12):ofab592. 10.1093/ofid/ofab592 34988246 PMC8694200

[13] Ho EC Cotter JM Thomas J Birkholz M Dominguez SR. Factors associated with actionable gastrointestinal panel results in hospitalized children. Hosp Pediatr. (2023) 13(12):1115–23. 10.1542/hpeds.2023-007273 37936503 PMC11318089

[14] Friesema IHM de Boer RF Duizer E Kortbeek LM Notermans DW Norbruis OF Etiology of acute gastroenteritis in children requiring hospitalization in the Netherlands. Eur J Clin Microbiol Infect Dis Off Publ Eur Soc Clin Microbiol. (2012) 31(4):405–15. 10.1007/s10096-011-1320-0 21725865

[15] Klein EJ Boster DR Stapp JR Wells JG Qin X Clausen CR Diarrhea etiology in a Children’s Hospital Emergency department: a prospective cohort study. Clin Infect Dis Off Publ Infect Dis Soc Am. (2006) 43(7):807–13. 10.1086/507335 16941358

[16] Wiegering V Kaiser J Tappe D Weissbrich B Morbach H Girschick HJ. Gastroenteritis in childhood: a retrospective study of 650 hospitalized pediatric patients. Int J Infect Dis IJID Off Publ Int Soc Infect Dis. (2011) 15(6):e401–407. 10.1016/j.ijid.2011.02.006 21489842

[17] Sameer M Masood A Almutawea L Fox G Loni R Ahmed A Gastrointestinal Panel Performance for the Diagnosis of Acute Gastroenteritis in Pediatric Patients. Cureus (2024). Available online at: https://www.cureus.com/articles/251150-gastrointestinal-panel-performance-for-the-diagnosis-of-acute-gastroenteritis-in-pediatric-patients (accessed October 7, 2025). 10.7759/cureus.61979 PMC11231452 38983994

[18] Shane AL Mody RK Crump JA Tarr PI Steiner TS Kotloff K 2017 Infectious Diseases Society of America clinical practice guidelines for the diagnosis and management of infectious diarrhea. Clin Infect Dis Off Publ Infect Dis Soc Am. (2017) 65(12):e45–80. 10.1093/cid/cix669 PMC5850553 29053792

[19] Yoo IH Kang HM Suh W Cho H Yoo IY Jo SJ Quality improvements in management of children with acute diarrhea using a multiplex-PCR-based gastrointestinal pathogen panel. Diagnostics. (2021) 11(7):1175. 10.3390/diagnostics11071175 34203426 PMC8303787

[20] Bizot E Bonacorsi S Labé P Pinhas Y Cointe A Ferroni A Use of gastrointestinal syndromic multiplex molecular assays and detection of Escherichia coli pathotypes in pediatric wards. J Clin Microbiol. (2025) 63(4):e01073–24. 10.1128/jcm.01073-24 40008873 PMC11980392

[21] Schutze GE Willoughby RE, Committee on Infectious Diseases, American Academy of Pediatrics. Clostridium difficile infection in infants and children. Pediatrics (2013) 131(1):196–200. 10.1542/peds.2012-2992 23277317

[22] Xie J Kim K Berenger BM Chui L Vanderkooi OG Grisaru S Comparison of a rapid multiplex gastrointestinal panel with standard laboratory testing in the management of children with hematochezia in a pediatric emergency department: randomized controlled trial. Microbiol Spectr. (2023) 11(3):e00268–23. 10.1128/spectrum.00268-23 37039648 PMC10269456

